# Systematic analysis of the lysine malonylome in common wheat

**DOI:** 10.1186/s12864-018-4535-y

**Published:** 2018-03-20

**Authors:** Jiabin Liu, Guangyuan Wang, Qi Lin, Wenxing Liang, Zhiqiang Gao, Ping Mu, Guiquan Li, Limin Song

**Affiliations:** 1 College of Agriculture, Shanxi Agricultural University, Taigu, Shanxi 030801 China; 2 Mordern Agriculture Demonstration Farm, Qindao Agricultural University, Qingdao, 266109 China; 3 College of Life Sciences, Qingdao Agricultural University, Qingdao, 266109 China; 4 College of Agronomy and Plant Protection, Qingdao Agricultural University, Qingdao, Shandong 266109 China

**Keywords:** Malonylome, Lysine malonylation, *Triticum aestivum* L., Calvin cycle, Post-translational modification

## Abstract

**Background:**

Protein lysine malonylation, a newly discovered post-translational modification (PTM), plays an important role in diverse metabolic processes in both eukaryotes and prokaryotes. Common wheat is a major global cereal crop. However, the functions of lysine malonylation are relatively unknown in this crop. Here, a global analysis of lysine malonylation was performed in wheat.

**Results:**

In total, 342 lysine malonylated sites were identified in 233 proteins. Bioinformatics analysis showed that the frequency of arginine (R) in position + 1 was highest, and a modification motif, K^ma^R, was identified. The malonylated proteins were located in multiple subcellular compartments, especially in the cytosol (45%) and chloroplast (30%). The identified proteins were found to be involved in diverse pathways, such as carbon metabolism, the Calvin cycle, and the biosynthesis of amino acids, suggesting an important role for lysine malonylation in these processes. Protein interaction network analysis revealed eight highly interconnected clusters of malonylated proteins, and 137 malonylated proteins were mapped to the protein network database. Moreover, five proteins were simultaneously modified by lysine malonylation, acetylation and succinylation, suggesting that these three PTMs may coordinately regulate the function of many proteins in common wheat.

**Conclusions:**

Our results suggest that lysine malonylation is involved in a variety of biological processes, especially carbon fixation in photosynthetic organisms. These data represent the first report of the lysine malonylome in common wheat and provide an important dataset for further exploring the physiological role of lysine malonylation in wheat and likely all plants.

**Electronic supplementary material:**

The online version of this article (10.1186/s12864-018-4535-y) contains supplementary material, which is available to authorized users.

## Background

Post-translational modifications (PTMs) are dynamic and reversible modifications of proteins and have wide effects in broadening the range of functionality of these proteins [1–3]. Many functional groups, such as phospho, ubiquityl, acetyl, methyl, and malonyl groups, are able to be introduced by PTMs [4]. Among PTMs, protein malonylation is a newly discovered modification, playing an important role in modulating various cellular processes [4–6]. Through reversible addition of a malonyl group to lysine residues, protein malonylation regulates protein localization, enzymatic activity, protein stability and many other biochemical processes. Although Malonyl-CoA is considered to be one of the most common donors of malonyl group, the enzymes controlling the malonylation status of proteins are largely unknown [7, 8]. Sirt5, a member of the lysine deacetylases (KDACs), was found to be able to catalyze lysine demalonylation reaction in mammalian cells [7, 8]. Therefore, it is speculated that both protein acetylation and malonylation are reversibly regulated by lysine acetyltransferases (KATs) and KDACs, which are located to diverse cell compartments [7–9]. Similar to lysine acetylation, many malonylated proteins localized in the nucleus, cytoplasm, mitochondria and chloroplast have been identified [5, 8, 10–13], indicating that a wide variety of biological processes are potentially regulated by lysine malonylation.

In recent years, as a result of advancements in liquid chromatography-tandem mass spectrometry (LC-MS/MS), a large number of lysine-acetylated proteins have been identified [2]. However, compared to these acetylation profiles, only a few organisms have been studied with respect to lysine malonylation, including *Bacillus amyloliquefaciens* [4], *Escherichia coli* [5], *Saccharopolyspora erythraea* [10], cyanobacteria (*Synechocystis*) [11], rice [12], HeLa cells [9], and mice [7, 13].

Common wheat (*Triticum aestivum* L.) is one of the most important cereal crops in the world. Systematic analysis of the lysine acetylated [2] and succinylated proteins [3] in common wheat revealed that they were involved in a variety of signaling pathways in development and metabolism. As one PTM that occurs on a lysine residue and competes with succinylation and acetylation, protein malonylation was expected to play a very important role in multiple processes in common wheat. To test this hypothesis, we performed the first proteomics study of lysine malonylation in common wheat. In total, we identified 342 lysine malonylation sites in 233 proteins. The malonylated proteins were associated with diversified biological processes and were distributed in multiple compartments, including the cytosol, chloroplast, nucleus, mitochondria, plasma membrane, cytoskeleton, extracellular space and peroxisome. Importantly, we found that 30% of the malonylated proteins were located to the chloroplast, and further studies showed that these proteins play an important role in photosynthetic carbon fixation in common wheat. Comparative analyses of proteomic profiles among the malonylome, acetylome and succinylome suggest that these three PTMs can occur on the same lysine residues and may coordinately regulate the function of many proteins in common wheat. This systematic analysis provides a rich dataset for further exploring the physiological role of lysine malonylation in this cereal crop and likely all plants.

## Methods

### Protein extraction from common wheat

Qing Mai 6, a common wheat variety (*T. aestivum* L.) tolerant to salt stress, was used for lysine malonylome analysis in this research. The seedlings of Qing Mai 6 were grown in a greenhouse for 3 weeks [12] and the excised leaves were subjected to protein extraction as previously described [14]. In brief, the leaves were ground in liquid nitrogen followed by sonicating for three times on ice in lysis buffer containing 8 M urea (Sigma-Aldrich, Saint Louis, USA), 1% TritonX-100 (Sangon Biotech, Shanghai, China), 10 mM dithiothreitol (DTT) (Sigma-Aldrich, Saint Louis, USA), and 1% Protease inhibitor cocktail (Merck Millipore, Billerica, USA) [14]. After centrifugation at 20,000×g 4 °C for 20 min, proteins in the supernatant were precipitated with tricarboxylic acid (TCA) (Sigma-Aldrich, Saint Louis, USA) at − 20 °C for 2 h [3]. The precipitates were washed three times by cold acetone (Hannuo, Lanxi, China) and were then redissolved in buffer (8 M urea, 100 mM NH_4_CO_3_, pH 8.0) [3]. Protein concentration was determined using the standard Bradford method.

### Affinity enrichment of lysine malonylated peptides

For affinity enrichment, the wheat proteins were cleaved into peptides through tryptic digestion. The extracted proteins were firstly reduced with 10 mM DTT at 37 °C for 1 h and alkylated with 20 mM iodoacetamide (IAA) (Sigma-Aldrich, Saint Louis, USA) for 45 min, and were then digested by trypsin (Promega, Madison, USA) as described [2]. Trypsin was added at 1:50 and 1:100 trypsin-to-protein mass ratio for the first and the second digestion, respectively [15]. A high pH reverse-phase HPLC with Agilent 300Extend C18 column (5 μm particles, 4.6 mm ID, 250 mm length) (Agilent, Santa Clara, USA) was used to fractionate the resulting peptides into 80 fractions [15]. Thereafter, the separated peptides were combined into 6 fractions [15]. To enrich peptides with malonylation sites, the combined peptides dissolved in NETN buffer (100 mM NaCl (Sigma-Aldrich, Saint Louis, USA), 1 mM EDTA (Sigma-Aldrich, Saint Louis, USA), 50 mM Tris-HCl (Sigma-Aldrich, Saint Louis, USA), 0.5% NP-40 (Sigma-Aldrich, Saint Louis, USA), pH 8.0) were incubated with pan anti-malonyllysine antibody conjugated agarose beads (PTM Biolabs, Hangzhou, China) at 4 °C overnight with gentle shaking [2]. The bound peptides were eluted from the beads with 0.1% trifluoroacetic acid (TFA) (Sigma-Aldrich, Saint Louis, USA) after 4 times washing with NETN buffer and were then cleaned using C18 ZipTips (Merck Millipore, Billerica, USA) [13].

### LC-MS/MS analysis

LC-MS/MS analysis of the malonylation peptides was carried out as described [15–18]. Briefly, the peptides cleaned with C18 ZipTips were separated with a reversed-phase analytical column (ThermoFisher Scientific, Waltham, USA). The gradient was comprised of an increase from 6 to 22% solvent B (0.1% formic acid (Sigma-Aldrich, Saint Louis, USA) in 98% acetonitrile (Sigma-Aldrich, Saint Louis, USA)) for 24 min, 22 to 40% for 8 min and climbing to 80% in 5 min then holding at 80% for the last 3 min, all at a constant flow rate of 300 nl/min on an EASY-nLC 1000 UPLC system. The resulting peptides were analyzed by Q Exactive™ Plus hybrid quadrupole-Orbitrap mass spectrometer (ThermoFisher Scientific, Waltham, USA) [16]. Intact peptides and ion fragments were detected in the Orbitrap at a resolution of 70,000 and 17,500, respectively, with the NCE setting at 30 [16]. A data-dependent procedure that alternated between one MS scan followed by 20 MS/MS scans was applied for the top 20 precursor ions above a threshold ion count of 5E3 in the MS survey scan with 15.0 s dynamic exclusion. [16]. An electrospray voltage of 2.0 kV was employed to the LC-MS/MS analysis [16]. To generate MS/MS spectra, 5E4 ions were accumulated and automatic gain control (AGC) was used to prevent overfilling of the orbitrap [16]. For MS scans, the m/z scan range was 350 to 1800 [16]. Fixed first mass was set as 100 m/z [15–18].

### Database search

MaxQuant with integrated Andromeda search engine (v.1.5.1.8) was used to process the obtained MS/MS data [19, 20]. The collected tandem mass spectra were searched against *4565_PR_wheat* database concatenated with reverse decoy database [3]. Trypsin/P was specified as cleavage enzyme allowing up to 4 missing cleavages, 5 modifications per peptide and 5 charges; minimum peptide length was set at 7 [3]. Mass error was set to 10 ppm and 0.02 Da for precursor ions and fragment ions, respectively [3]. Carbamidomethylation on Cys was set as fixed modification; malonylation on Lys was set as variable modification [2]. False discovery rate (FDR) threshold of 1% was specified for protein, peptide and modification site [2]. All the other parameters in MaxQuant were set as default values [2]. The probability for site localization was set as > 0.75 [2, 15–18].

### Bioinformatics analysis

The malonylated proteins were analyzed by Gene Ontology (GO) annotation derived from the UniProt-GOA database (http://www.ebi.ac.uk/GOA/) based on biological process, cellular component and molecular function [13]. InterPro (http://www.ebi.ac.uk/interpro/) was used to annotate functional domains of all identified proteins [14]. Protein pathways were annotated by Kyoto Encyclopedia of Genes and Genomes (KEGG) database [21]. A two-tailed Fisher’s exact test was employed to test the enrichment of the malonylated proteins and a corrected *p*-value < 0.05 was considered significant [15]. Subcellular localization of the modified proteins was predicated with WoLF PSORT (version PSORT/PSORT II) [22]. The current dataset used to train WoLF PSORT contains over 12,000 animal sequences and more than 2000 plant and fungi sequences respectively. For each protein, WoLF PSORT reported a number to roughly indicate the number of nearest neighbors to the protein which localize to each site. Then, localization sites with the maximum number were selected as the subcellular localization of protein. The model of sequences constituted with amino acids in specific positions of modify-21-mers was analyzed using Motif-x software and the secondary structures of proteins were predicted using NetSurfP software [23]. Only predictions with a minimum probability of 0.5 for one of the different secondary structures (coil, α-helix, β-strand) were considered for analysis. The mean secondary structure probabilities of the malonylated lysine residues were compared with the mean secondary structure probabilities of a control dataset containing all the lysine residues of all the malonylated proteins identified in this study. Cytoscape software was employed to analyze the protein-protein interactions for the malonylated proteins and the protein-protein interaction network was obtained from the STRING database [24, 25]. BLASTP was carried out to determine the conservation of lysine malonylated proteins between common wheat and other organisms.

### Immunoprecipitation of dehydroascorbate reductase

The leaves of common wheat were ground in liquid nitrogen and the soluble proteins were extracted as previously described [3]. After overnight incubation of 1 mg of soluble proteins with or without 1 μg of dehydroascorbate reductase specific antibody (Agrisera, Vännäs, Sweden), 50 μl of protein A agarose beads (GE Healthcare, Uppsala, Sweden) were added and the solution was incubated for another 1 h. The bound proteins were eluted from the beads with boiled SDS-PAGE sample buffer following 5 times wash with lysis buffer [3].

### Western blot analysis

Eluted proteins were separated by SDS-PAGE on a 12% gel and probed with dehydroascorbate reductase antibody (1:5000 dilution) and anti-malonyllysine antibody (1:2000 dilution, PTM Biolabs, Hangzhou, China), respectively [3]. Proteins were visualized using Pierce Fast Western Blot Kit ECL Substrate (ThermoFisher Scientific, Waltham, USA) according to the manufacturer’s instructions [3].

## Results and discussion

### Identification of lysine malonylated proteins in common wheat

To elucidate the regulatory functions of lysine malonylation in wheat, a proteome-scale analysis of malonylated proteins was performed (Additional file 1: Figure S1a). To validate the MS data, the mass error of all the identified peptides was checked, and the total distribution of the mass error was less than 5 ppm, indicating that the mass accuracy of the MS data fit the requirement (Additional file 1: Figure S1b). We further checked the distribution of all the peptides, and the results showed that the length of most of the peptides was between 8 and 20 (Additional file 1: Figure S1c), which demonstrated that the sample preparation met the standard [15, 17]. After a large-scale lysine malonylome analysis using LC-MS/MS, a total of 342 lysine malonylation sites in 233 protein groups were identified (Additional file 2: Table S1). Three representative MS/MS spectra of the malonylated peptides were showed in Additional file 1: Figure S2. The number of malonylated proteins in common wheat was higher than that in *S. erythraea* [4], but less than the number of identified proteins in *B. amyloliquefaciens* [4], *E. coli* [5] and mammals [7, 13]. Dehydroascorbate reductase (DHAR), a protein involved in redox homeostasis under biotic and abiotic stresses and a key component of the ascorbate recycling system, was found to be malonylated on the lysine residue, K157 (Additional file 2: Table S1). Immunoprecipitation coupled with Western blot analysis confirmed malonylation of DHAR in wheat leaves (Additional file 1: Figure S3). Proteins can be modified on either one or several amino acid residues. Thus, the number of malonylated sites in the identified proteins was calculated in common wheat. As shown in Additional file 1: Figure S4, 75% (175) of malonylated proteins had only one malonylated lysine site, whereas 25% (58) were modified on multiple lysine residues. A similar distribution pattern was also found in *S. erythraea* [10]. To our knowledge, these findings represent the first report of lysine malonylome in common wheat.

### Pattern analysis of malonylated sites

**Fig. 1:**
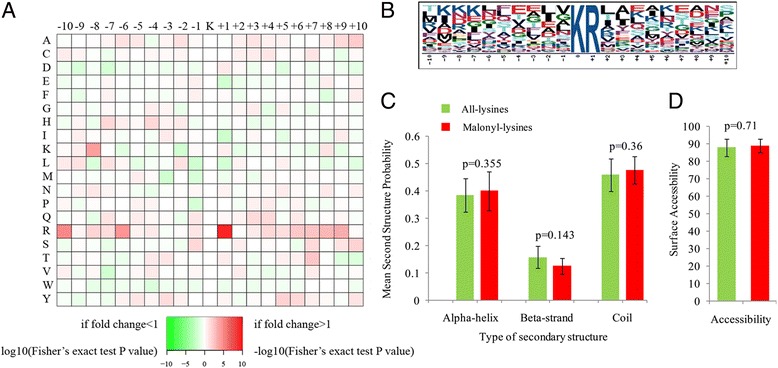
Properties of the lysine malonylation peptides in common wheat. **a** Heat map of the amino acid compositions of the malonylation sites showing the frequency of different amino acids around the modified lysine. Red indicates high frequency and green means low frequency. **b** Malonylation sequence motifs for ±10 amino acids around the lysine malonylation sites. **c** Probabilities of lysine malonylation in the structures of alpha-helix, beta-strand and coli. **d** Predicted surface accessibility of malonylation sites

Previous studies have demonstrated the modified site preferences for lysine residues at particular positions [10, 15, 17, 18]. To map the specific amino acids surrounding the malonylated lysine, we investigated the frequency of different amino acids around the malonylated lysine from − 10 to + 10. As shown in Fig. 1a, the frequency of arginine (R) in position + 1 was highest. We further investigated the sequence motifs in all the identified peptides using the motif-x program. In accordance with the heat map of the amino acid compositions of the malonylation sites (Fig. 1a), a motif, K^ma^R (K^ma^ indicates the lysine malonylation site), was obtained (Fig. 1b). In addition to K^ma^R, K(X6)K^ma^, K^ma^(X6)K, and K(X5)K^ma^ (X represents an unspecified amino acid residue) were also detected in *S. erythraea* [10]. However, compared with acetylation motifs [2, 16, 18] and succinylation motifs [26, 27], the number of lysine malonylation motifs identified so far was much lower.

To elucidate a possible relationship between lysine malonylation sites and protein structure in wheat, we performed a structural analysis of all the identified proteins. Consistent with the results in *B. amyloliquefaciens* [4], the malonylation sites do not prefer a certain type of secondary structure such as an alpha-helix or beta-strand (*P* > 0.05) (Fig. 1c), indicating that there is no tendency toward malonylation in common wheat. We further evaluated the surface accessibility of the lysine malonylation sites. As shown in Fig. 1d, compared to 87.48% of all lysine residues, 88.71% of lysine malonylation sites were exposed to a protein surface (*P* = 0.71), indicating that the surface property of proteins is not affected by lysine malonylation. Different from our observations, the lysine malonylation sites were preferably located on the surface of proteins in *B. amyloliquefaciens* [4].

### Conservation of lysine malonylated proteins

**Fig. 2:**
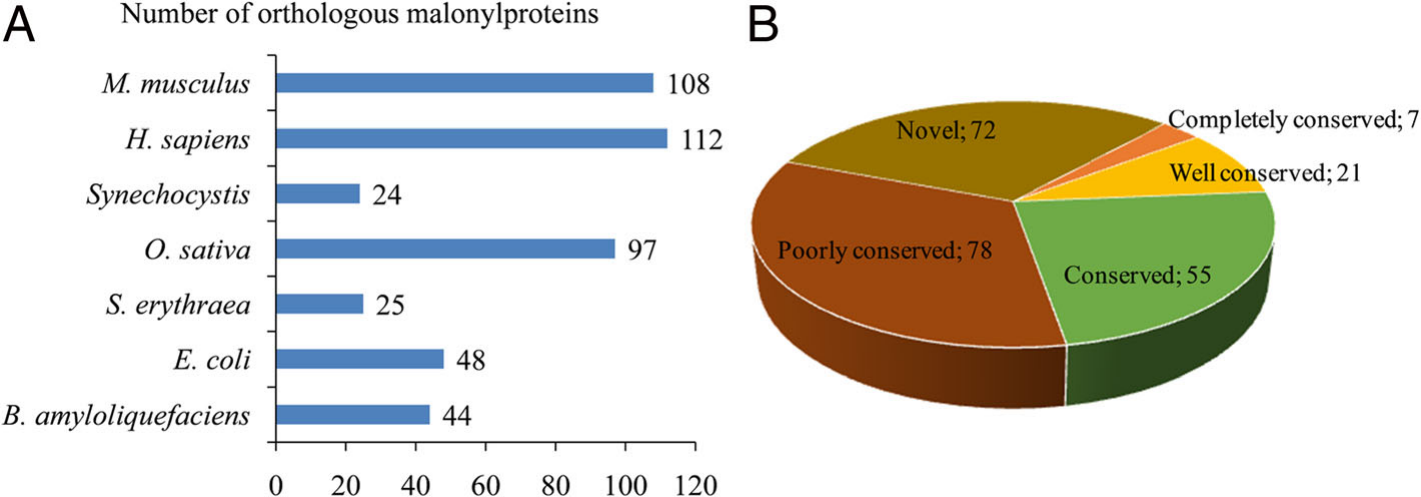
Conservation analysis of lysine malonylated proteins. **a** Number of orthologous malonylproteins in seven organisms with reported malonylomes. **b** A pie chart of conservation of malonylproteins in *B. amyloliquefaciens*, *E. coli*, *H. sapiens*, *M. musculus*, *O. sativa*, *S. erythraea* and *Synechocystis*. Grouping was performed as follows: Completely conserved, 7 orthologs; Well conserved, 5 to 6 orthologs; Conserved, 3 to 4 orthologs; Poorly conserved, 1 to 2 orthologs; Novel, 0 orthologs

To date, a large number of lysine malonylated proteins have been identified in several prokaryotes and eukaryotes [4, 5, 7, 10–12]. However, the conservation of lysine malonylation in these organisms is unknown. As such, we searched the orthologs of lysine malonylated proteins in common wheat by a protein BLAST search against seven organisms with determined malonylomes: *B. amyloliquefaciens*, *E. coli*, *Homo sapiens*, *Mus musculus*, *O. sativa*, *S. erythraea* and *Synechocystis* sp. PCC 6803*.* Totally, 458 orthologs of the malonylproteins in common wheat were identified in these seven organisms (Additional file 2: Table S2). As shown in Fig. 2a, 161 malonylated proteins have orthologs in *M. musculus* (108 proteins), *H. sapiens* (112 proteins), *Synechocystis* (24 proteins), *O. sativa* (97 proteins), *S. erythraea* (25 proteins), *E. coli* (48 proteins) and *B. amyloliquefaciens* (44 proteins), which account for 69.1% (161/233 proteins) of the total malonylproteins in common wheat. We further classified the malonylated proteins of common wheat depending on the number of their orthologous proteins in other organisms. The results showed that the percentage of completely conserved proteins (have orthologs in all 7 organisms), well-conserved proteins (have orthologs in 5 to 6 organisms), conserved proteins (have orthologs in 3 to 4 organisms) and poorly conserved proteins (have orthologs in 1 to 2 organisms) were 3% (7/233 proteins), 9% (21/233 proteins), 23.6% (55/233 proteins), and 33.5% (78/233 proteins) (Fig. 2b), respectively. Furthermore, 30.9% (72/233 proteins) of the malonylated proteins in common wheat was grouped as novel proteins since no orthologs were identified in other organisms (Fig. 2b). These observations suggest that although lysine malonylation is largely conserved in prokaryotes and eukaryotes, different organisms contain unique malonylated proteins with specific functions.

### Functional annotation of malonylated proteins

**Fig. 3:**
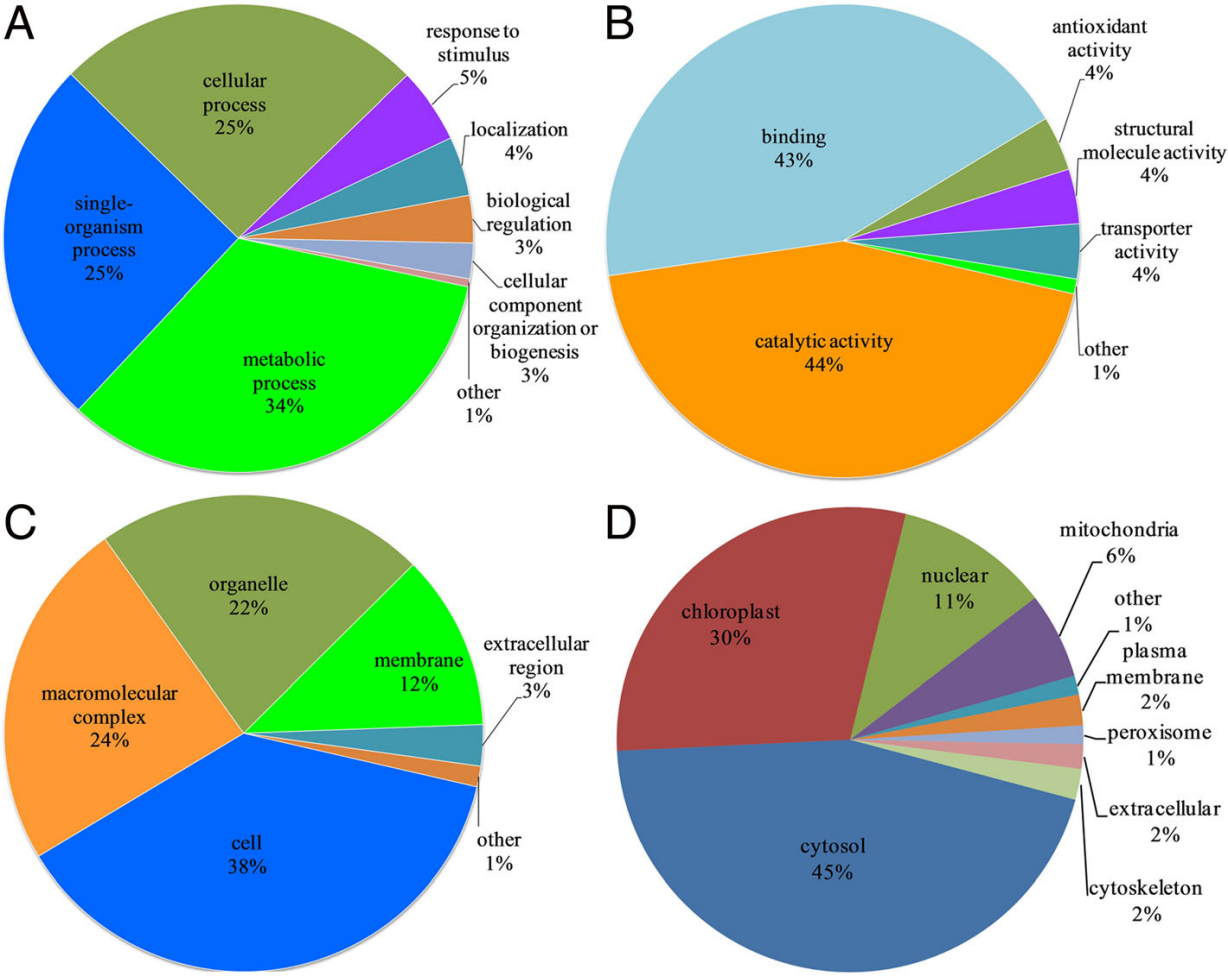
Functional classification of lysine malonylated proteins in common wheat. **a** Classification of malonylated proteins according to biological process. **b** Classification of malonylated proteins according to cellular component. **c** Classification of malonylated proteins according to molecular function. **d** Subcellular localization of the malonylated proteins

To further understand the potential roles of malonylated proteins, we performed a GO functional classification analysis in common wheat based on their biological process, cellular component and molecular function. As shown in Fig. 3a and Additional file 2: Table S3, according to biological process analysis, 123 proteins in metabolic processes, 93 proteins in single-organism processes, and 93 proteins in cellular processes were malonylated, which account for 34, 25 and 25% of all identified proteins in common wheat, respectively. Consistent with these observations, a large proportion of the malonylated proteins were associated with catalytic (44%) and binding (43%) activities based on molecular function analysis (Fig. 3b, Additional file 2: Table S3). Cellular component analysis showed that most of the malonylated proteins were located in the cell (38%), macromolecular complex (24%), organelle (22%), membrane (12%) and extracellular region (3%) (Fig. 3c, Additional file 2: Table S3). These results suggest that lysine malonylation may affect the molecular functions of proteins and then regulate multiple biological processes in diverse cellular components in common wheat.

We further predicted the subcellular localizations of the identified proteins using WoLF PSORT [22]. The results in Fig. 3d showed that most of the malonylated proteins in wheat were located in the cytosol (45%) and chloroplast (30%). Interestingly, 11% of the malonylated proteins were localized in the nucleus, including histone H4, histone H2B, histone H2A, and histone H3 (Fig. 3d, Additional file 2: Table S1), confirming the regulatory role of lysine malonylation in post-translational regulation. Furthermore, other malonylated proteins were predicted to be located to the mitochondria (6%), plasma membrane (2%), cytoskeleton (2%), extracellular space (2%) and peroxisome (1%) (Fig. 3d). These findings indicate that the lysine-malonylated proteins have a broad range of biological functions in common wheat.

### Functional enrichment analysis of malonylated proteins

**Fig. 4:**
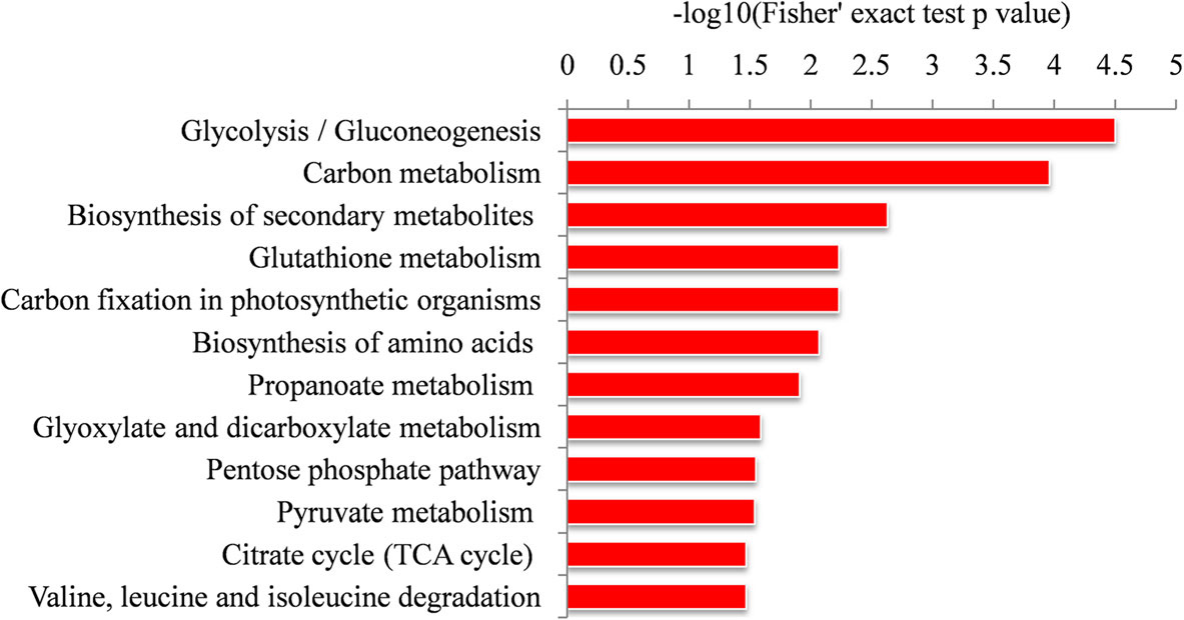
KEGG pathway-based enrichment analysis of the malonylation proteins in common wheat

To evaluate the nature of malonylated proteins in wheat, we performed functional enrichment analyses of the GO, KEGG pathway and protein domain. As shown in Additional file 1: Figure S5 and Additional file 2: Table S4, many modified proteins were associated with the metabolic and catabolic processes in the biological process category. In agreement with these observations, most of the malonylated proteins were found to be involved in enzymatic activities and binding according to the enrichment analysis of molecular function (Additional file 1: Figure S5 and Additional file 2: Table S4). Consistent with these findings, proteins located to the cytoplasm, macromolecular complex, and DNA bending complex were more likely to be malonylated based on the enrichment analysis of the cellular component (Additional file 1: Figure S5 and Additional file 2: Table S4). In support of these findings, the proteins associated with carbon metabolism, secondary metabolite biosynthesis, carbon fixation in photosynthetic organisms, and amino acid biosynthesis were highly enriched based on the KEGG pathway enrichment analysis (Fig. 4 and Additional file 2: Table S5). Moreover, protein domain enrichment analysis showed that proteins with the following domains were more prone to malonylation: NAD(P)-binding, GTP-binding, catalytic and transferase domains (Additional file 1: Figure S6 and Additional file 2: Table S6).

### Protein interaction network of malonylated proteins

**Fig. 5:**
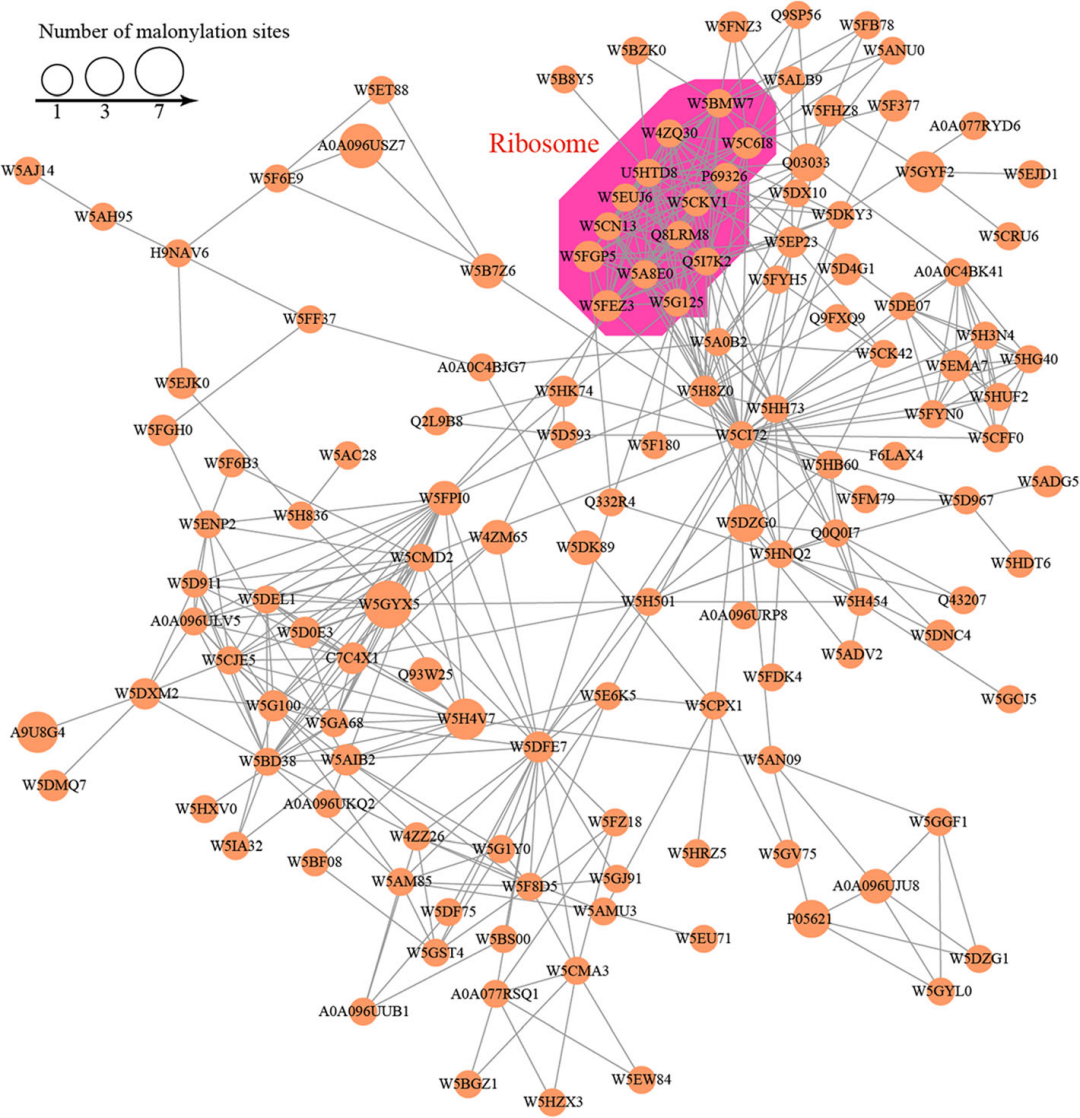
Protein interaction networks of the malonylation proteins in common wheat

To investigate the cellular processes regulated by lysine malonylation in wheat, a protein interaction network was established with the STRING database using Cytoscape software [24]. As shown in Fig. 5 and Additional file 2: Table S7, 137 malonylated proteins were mapped to the protein network database. According to the algorithm in the Cytoscape software, eight highly interconnected clusters of acetylated proteins were retrieved. The top cluster identified included proteins associated with ribosomes (Additional file 1: Figure S7). The complicated interaction networks of malonylated proteins indicate that a variety of pathways are putatively modulated by lysine malonylation in common wheat.

### Analysis of malonylated proteins involved in the Calvin cycle

**Fig. 6:**
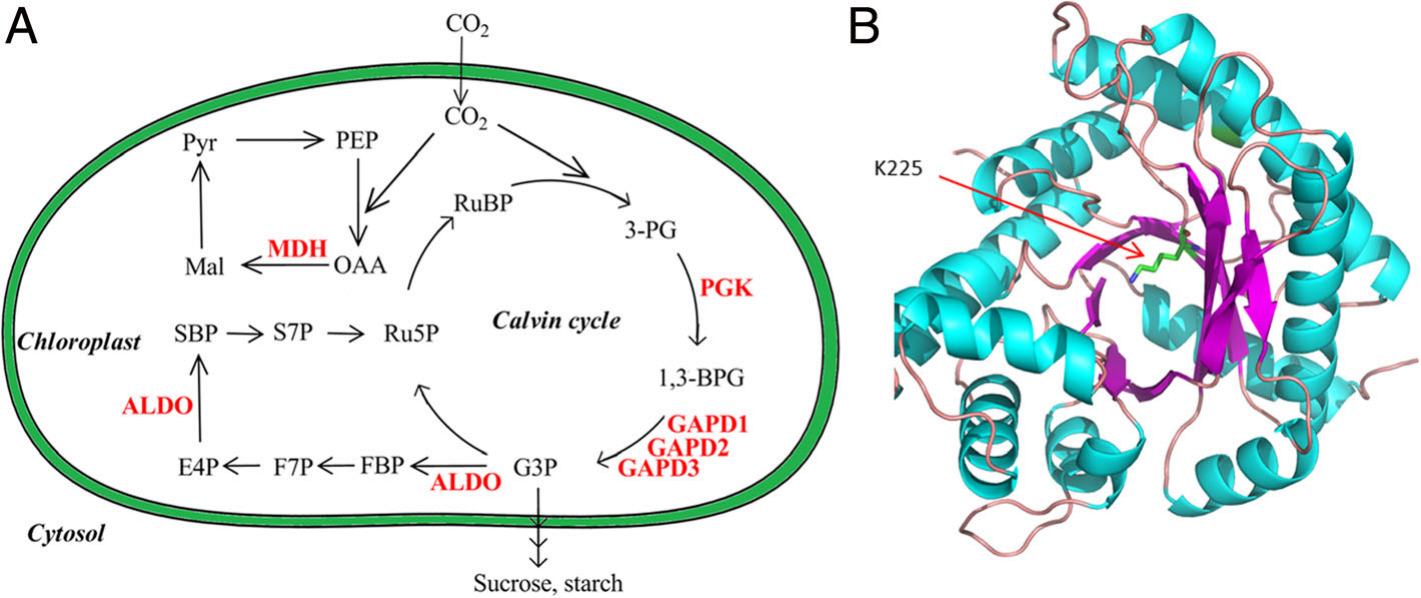
Identified malonylation proteins in Calvin cycle in common wheat. **a** Working scheme of lysine malonylation events involved in Calvin cycle in common wheat. The malonylated proteins were highlighted in red. **b** Three-dimensional structure of protein ALDO with identified malonylation site. The structure was taken from PDB database. The malonylated lysine residue, K225, was indicated by red arrow

In wheat, the Calvin cycle is one of the most important metabolic processes. Previous studies demonstrated that carbohydrate metabolism in Calvin cycle is regulated by drought stress in wheat [28].The Calvin cycle (also known as the Calvin-Benson cycle) is the set of chemical reactions that convert carbon dioxide into carbohydrates and other organic compounds [29, 30]. The Calvin cycle occurs in chloroplasts during photosynthesis. We found that 30% of the malonylated proteins were located in the chloroplast in common wheat (Fig. 3d). Functional enrichment analysis of the KEGG pathway revealed that the proteins associated with carbon fixation in photosynthetic organisms were highly enriched (Fig. 4). These results suggested that lysine malonylation may play an important role in the Calvin cycle in common wheat. To confirm these observations, we further analyzed the malonylated proteins involved in the Calvin cycle. In agreement with our hypothesis, six enzymes involved in carbon fixation in the Calvin cycle were found to be malonylated, including phosphoglycerate kinase (PGK) (accession no. W5H4V7), glyceraldehyde 3-phosphate dehydrogenase (GAPD1–3) (accession nos. W5GYX5, C7C4X1 and W5G5H3), fructose-bisphosphate aldolase (ALDO) (accession no. W5D0E3), and malate dehydrogenase (MDH) (accession no. W5AIB2) (Fig. 6a, Additional file 2: Table S1). Importantly, all the malonylated sites were located in the functional domains of these enzymes (Additional file 2: Table S1). An example is ALDO carrying the malonylated K225, which is the catalytic site of this enzyme (Fig. 6b). Although ribulose-1.5-bisphosphate carboxylase/oxygenase (RuBisCO), a central enzyme in the Calvin cycle, was detected as a differentially expressed protein in response to drought stress [28], this protein was not modified by malonylation. These results suggest that lysine malonylation probably modulates the Calvin cycle in common wheat.

### Comparison among the malonylome, acetylome and succinylome in common wheat

**Fig. 7:**
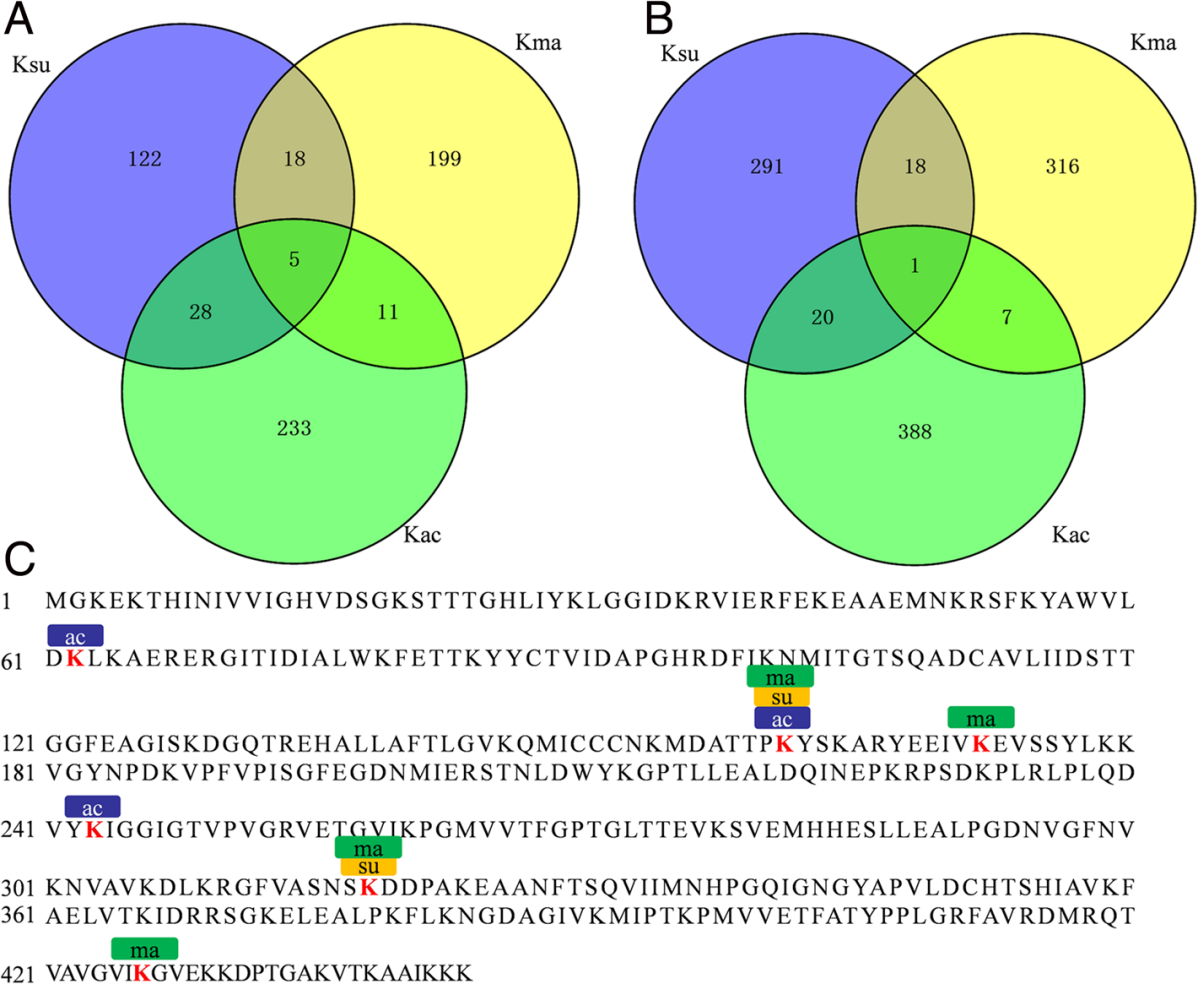
Overlap among lysine malonylation, lysine succinylation and lysine acetylation in common wheat. **a** Overlap of malonylated proteins, succinylated proteins and acetylated proteins. **b** Overlap of malonylation sites, succinylation sites and acetylation sites. **c** A representative protein, elongation factor 1-alpha (accession no. Q03033), showing the overlap among malonylation, succinylation and acetylation

As one type of PTM, lysine malonylation competes with other modifications, such as succinylation and acetylation, for lysine residues [5, 10, 31]. In our previous research, 416 acetylated lysine sites in 277 proteins [2] and 330 lysine succinylated modification sites in 173 proteins [3] were identified. To determine whether malonylation, acetylation and succinylation can occur on the same lysine residue, we compared the lysine malonylome with the succinylome and acetylome. As shown in Fig. 7a, 5 proteins were modified by the three PTMs at the same time, including GAPD1 (accession no. W5GYX5), which is one of the key enzymes involved in the Calvin cycle [29, 30]; elongation factor 1-alpha (accession no. Q03033), which is responsible for the transport of amino acyl tRNAs to ribosomes [32]; 14–3-3 protein (accession no. L0GED8), which is a negative regulator in response to nutrient sensing [33]; translationally controlled tumor protein homolog (accession no. Q8LRM8); and 4-hydroxy-7-methoxy-3-oxo-3,4-dihydro-2H-1,4-benzoxazin-2-yl glucoside beta-D-glucosidase 1a (accession no. Q1XIR9) (Additional file 2: Table S1). A total of 16 proteins were modified by both lysine malonylation and acetylation, and 23 proteins were modified by both lysine malonylation and succinylation in wheat (Fig. 7a). Moreover, lysine acetylation and succinylation can simultaneously happen on 33 proteins (Fig. 7a).

We further compared the lysine residues modified by lysine malonylation, succinylation and acetylation in wheat [2, 3]. As shown in Fig. 7b, 2.3 and 5.6% of the malonylation sites could also be acetylated and succinylated, respectively. Only one lysine site in elongation factor 1-alpha (accession no. Q03033) was simultaneously modified by these three types of modifications (Fig. 7b, Additional file 2: Table S1**)** [2, 3]. The overlap among malonylation, acetylation and succinylation in elongation factor 1-alpha was shown in Fig. 7c. Four malonylated sites at K161, K172, K318 and K427 were identified, and two sites at K161 and K318 were also determined to be succinylated. Moreover, three lysine sites at K62, K161, and K243 were found to be acetylated (Fig. 7c).

Multiple PTMs, including malonylation, succinylation, acetylation and phosphorylation, also occur on the same proteins in *E. coli* [5] and *S. erythraea* [10]. A total of 594 lysine malonylation sites were able to be modified by succinylation and acetylation in *E. coli* [5]. In *S. erythraea*, 16 proteins were found to be modified by malonylation, acetylation and phosphorylation at the same time [10]. These results, together with our findings, indicate that multiple PTMs can occur on the same proteins to coordinately regulate the function of many proteins in common wheat and likely all eukaryotic and prokaryotic organisms.

## Conclusions

In this study, a total of 233 malonylated proteins with 342 unique modification sites were identified in common wheat. The identified malonylated proteins are localized in diverse compartments and are involved in multiple biological processes, especially in the Calvin cycle. Protein interaction network analysis demonstrates that numerous pathways are modulated by lysine malonylation. Overlap among the acetylome, succinylome and malonylome in wheat shows coordination among these three important PTMs. The provided dataset illuminates a crucial role of lysine malonylation in common wheat and serves as an important resource for examining and exploring the physiological role of lysine malonylation in all plants.

## Additional files

Additional file 1: **Figure S1.** Systematic analysis of lysine malonylation sites in common wheat. **Figure S2.** Three representative MS/MS spectra of the malonylated peptides. **Figure S3.** Validation of lysine malonylation of DHAR by Western blot analysis. **Figure S4.** Number of malonylation sites per protein in common wheat. **Figure S5.** GO-based enrichment analysis in terms of cellular component (red bars), molecular function (blue bars) and biological process (green bars). **Figure S6.** Domain-based enrichment analysis of malonylated proteins. **Figure S7.** Interaction network of malonylated proteins associated with ribosome. (DOCX 2050 kb)

Additional file 2: **Table S1.** The identified lysine malonylation sites in wheat. **Table S2** Conservation of lysine malonylated proteins. **Table S3** The distribution of proteins in GO terms. **Table S4** Protein GO enrichment based on cellular component, molecular function and biological process. **Table S5** Protein pathway enrichment analysis. **Table S6** Protein domain enrichment analysis. **Table S7** Protein interaction network of identified proteins. (XLSX 141 kb)

## Abbreviations

AGC: Automatic gain control
ALDO: Fructose-bisphosphate aldolase
DHAR: Dehydroascorbate reductase
DTT: Dithiothreitol
FA: Formic acid
FDR: False discovery rate
GAPD: Glyceraldehyde 3-phosphate dehydrogenase
GO: Gene ontology
HPLC: High performance liquid chromatography
IAA: Iodoacetamide
KEGG: Kyoto encyclopedia of genes and genomes
LC − MS/MS: Liquid chromatography-mass spectrometry
MDH: Malate dehydrogenase
PGK: Phosphoglycerate kinase
PTM: Post-translational modification
TCA: Tricarboxylic acid
TFA: Trifluoroacetic acid
