# Supplementary material for: Systematic analysis of the lysine malonylome in common wheat
Source: BMC Genomics. 2018 Mar 20;19:209. doi: 10.1186/s12864-018-4535-y (PMC5859436; doi:10.1186/s12864-018-4535-y)
Supplement: Supplementary file 1 — Figure S1. Systematic analysis of lysine malonylation sites in common wheat. Figure S2. Three representative MS/MS spectra of the malonylated peptides. Figure S3. Validation of lysine malonylation of DHAR by Western blot analysis. Figure S4. Number of malonylation sites per protein in common wheat. Figure S5. GO-based enrichment analysis in terms of cellular component (red bars), molecular function (blue bars) and biological process (green bars). Figure S6. Domain-based enrichment analysis of malonylated proteins. Figure S7. Interaction network of malonylated proteins associated with ribosome. (DOCX 2050 kb) [file 12864_2018_4535_MOESM1_ESM.docx]

**Systematic analysis of the lysine malonylome in common wheat**

Jiabin Liu^1, 2^, Guangyuan Wang^3^, Qi Lin^4^, Wenxing Liang^4^, Zhiqiang Gao^1^, Ping Mu^4^, Guiquan Li^1*^, Limin Song^4*^

**Figure S1.** Systematic analysis of lysine malonylation sites in common wheat.

**Figure S2.** Three representative MS/MS spectra of the malonylated peptides.

**Figure S3.** Validation of lysine malonylation of DHAR by Western blot analysis.

**Figure S4.** Number of malonylation sites per protein in common wheat.

**Figure S5.** GO-based enrichment analysis in terms of cellular component (red bars), molecular function (blue bars) and biological process (green bars).

**Figure S6.** Domain-based enrichment analysis of malonylated proteins.

**Figure S7.** Interaction network of malonylated proteins associated with ribosome.

**Figure S1.** Systematic analysis of lysine malonylation sites in common wheat. **a** Overview of experimental procedures used in this study. **b** Mass error distribution of all identified peptides. **c** Peptide length distribution.


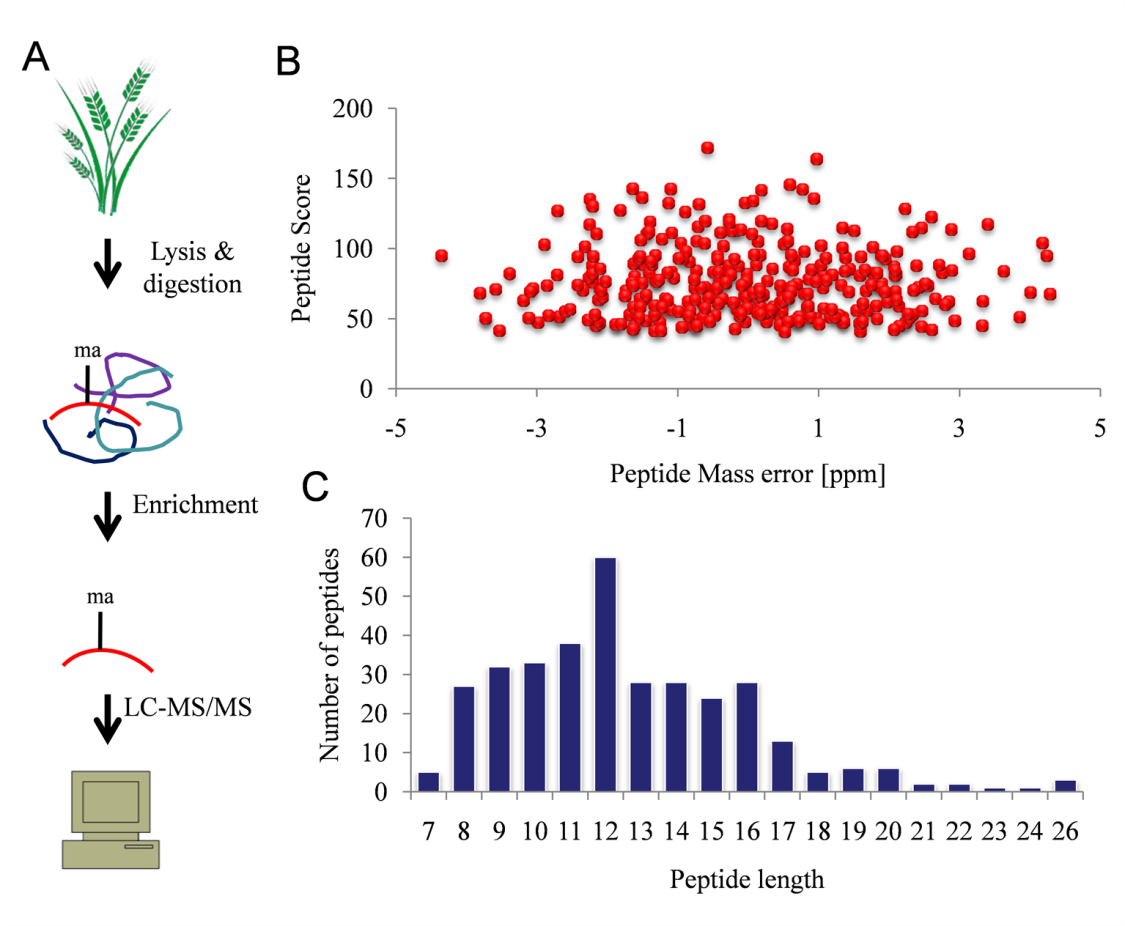


**Figure S2.** Three representative MS/MS spectra of the malonylated peptides. The peptides were from phosphoglycerate kinase (W5H4V7), fructose-bisphosphate aldolase (W5D5L4), proteasome subunit alpha type (W5H3N4), respectively. The malonylated (ma) lysine residues, 129, 38 and 115 were indicated.


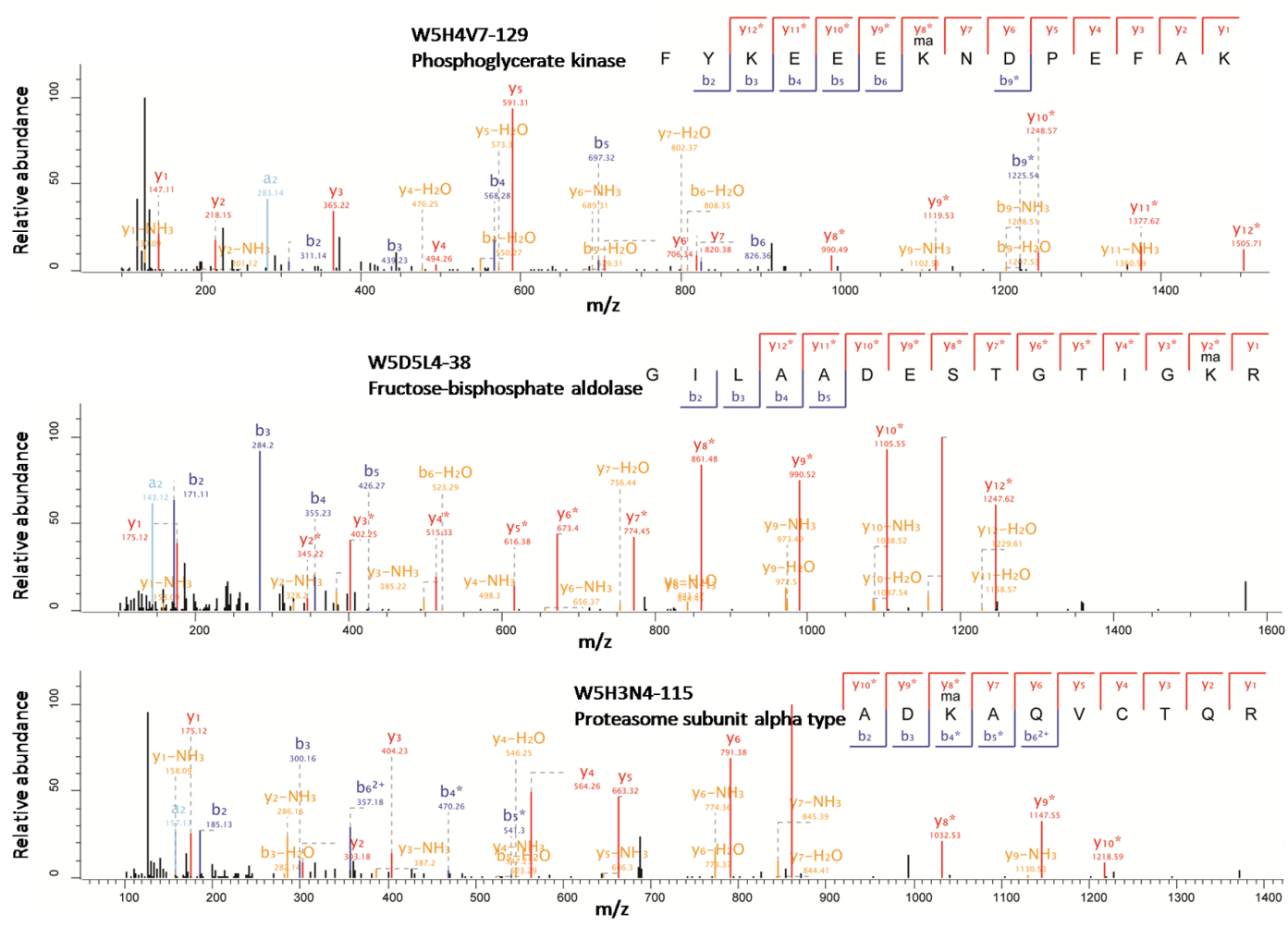


**Figure S3.** Validation of lysine malonylation of DHAR by Western blot analysis. Immunoprecipitation of DHAR was performed with (+) or without (-) DHAR antibody (Ab) and the eluted proteins were probed with either anti-malonyl lysine antibody (maK) or DHAR antibody.

**Figure S4.** Number of malonylation sites per protein in common wheat. Proteins with one, two, three, four and more malonylated sites were shown in green, blue, yellow and red, respectively. Percentages of these proteins were indicated.

**
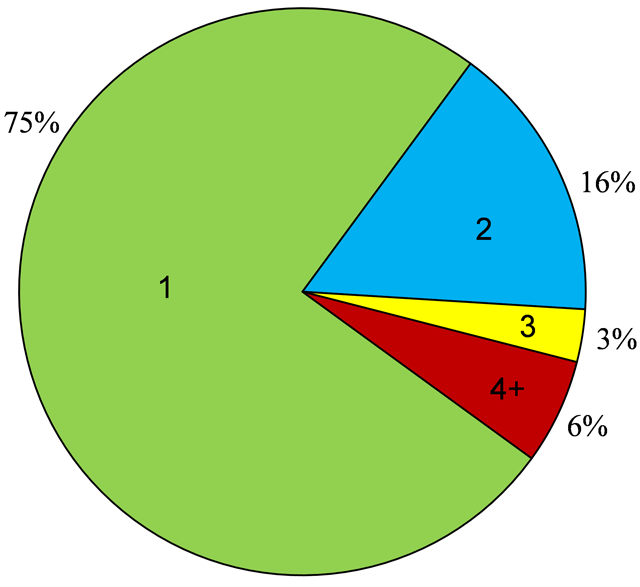
**

**Figure S5.** GO-based enrichment analysis in terms of cellular component (red bars), molecular function (blue bars) and biological process (green bars).

**Figure S6.** Domain-based enrichment analysis of malonylated proteins.

**Figure S7.** Interaction network of malonylated proteins associated with ribosome.
